# Ten years of vaccinovigilance in Italy: an overview of the pharmacovigilance data from 2008 to 2017

**DOI:** 10.1038/s41598-020-70996-x

**Published:** 2020-08-24

**Authors:** F. Moretti, L. Gonella, S. Gironi, A. R. Marra, C. Santuccio, P. Felicetti, F. Petronzelli, P. Marchione, S. A. Barnaba, A. Poli, G. Zanoni, U. Moretti

**Affiliations:** 1 Section of Hygiene and Environmental Occupational Preventive Medicine, Department of Diagnostics and Public Health, University of Verona, Verona, Italy; 2 Section of Pharmacology, Department of Diagnostics and Public Health, University of Verona, Verona, Italy; 3 Italian Medicines Agency, Rome, Italy; 4 Local Health Unit (ASL), Rieti, Italy; 5 Immunology Unit, University Hospital, Verona, Italy

**Keywords:** Drug regulation, Public health

## Abstract

Reporting and analysis of Adverse Events Following Immunization (AEFIs) are the cornerstones of vaccine safety surveillance prompting causality assessment and signal detection. This paper describes the impact of the Italian Pharmacovigilance System of vaccines over a 10-year period (2008–2017). The reporting rate (RR) per all distributed dose was calculated. Serious AEFIs and causality assessments for fatal cases were described. The main results from signal detection were reported. During the study period, 46,430 AEFIs were reported with an overall RR of 17.2 per 100,000 distributed doses. Italy showed the highest number of reports among European countries. Only 4.4% of the reports came from citizens. Of the total, 12.7% were classified as serious with a RR over the study period of 2.20 per 100,000 distributed doses. They were mainly related to hyperpyrexia and usually had a positive outcome. Fatal outcomes were reported in 0.3% of the cases and were primarily associated with the influenza vaccine in elderly patients. None of these outcomes had a consistent causal association with the vaccination. Febrile convulsions by the measles, mumps, rubella and varicella vaccines and intussusception by the rotavirus vaccine were among the highlighted signals. The reporting rate and the analysis of serious events from 10 years support the good risk/benefit profiles of vaccines.

## Introduction

Vaccination is one of the most important achievements in public health. It prevented at least 10 million deaths between 2010 and 2015^1^. Despite substantial evidence for the benefits of immunization, they may not be immediately perceived by the general population. Moreover, misconceptions about vaccine safety still influence public confidence and adherence to immunization programmes. In fact, safety concerns are the main reason for vaccination refusal in Western countries^2^. Nevertheless, the risks of adverse events following immunization (AEFIs) are very low compared with those associated with the target diseases^3–6^.

Low public confidence in the safety of immunization programmes may impede vaccination coverage and compromise herd immunity protection. This effect has serious consequences for population^7,8^. A dramatic recent example was the decline in measles vaccination coverage in Italy. It caused a large outbreak since January 2017 which accounted for 29% of all measles cases reported by EU countries in 2017–2018^9^. At the same time, poliomyelitis vaccine coverage fell below the 95% threshold. Consequently, in June 2017, the Italian Ministry of Health increased the number of compulsory vaccinations from 4 to 10 (diphtheria, tetanus, hepatitis B, poliomyelitis, pertussis, *Haemophilus influenzae* type b, measles, mumps, rubella, and varicella) for children up to 16 years of age (Law no 119/2017). This policy had a positive impact on all vaccination coverage^10^.

The new legislation increased public awareness about the importance of vaccines and vaccination. However, parental adherence to vaccination programmes is complex and requires further evaluation^11^. The EMA Guideline on Good Pharmacovigilance Practices (GVP) states that a significant factor affecting public vaccine confidence is the knowledge that systems are in place to ensure complete and rapid assessment and to take precautionary measures if needed^12^.

The two main goals of an effective vaccinovigilance system are to: (1) monitor and evaluate vaccine safety through early and timely detection and management of any AEFIs to ensure favourable risk/benefit profiles and the safe use of vaccines, and (2) ensure transparent and up-to-date communication of vaccine risk/benefit profiles and provide information and answers to the public to allay their vaccine safety concerns. Vaccinovigilance, then, can effectively contend with the “social amplification of risk” phenomenon and foster a vaccination culture by increasing public confidence in it^13^.

The foundation of any safety surveillance system is timely reporting. These systems can trigger safety signals or awareness of new or known adverse events requiring further investigation. The purpose of the changes made to the European Pharmacovigilance (PhV) Legislation of 2012 was to increase the transparency and efficiency of relevant activities, to enhance patient/consumer participation in the PhV system, and to promote incident reporting using various tools, such as the internet^14,15^. Vaccinovigilance in the Italian PhV system was presented in the form of a survey of 26 European countries^16^. It was also briefly described in 2015^17^. To date, however, very little has been published on the Italian vaccinovigilance system. Studies describing national vaccinovigilance data have been published for Australia^18^, China^19^, Oman^20^, Switzerland^21^, and the United States^22^. Other vaccinovigilance data have been reported for specific regions such as Veneto, Italy^23^, Valencia, Spain^24^, and Rondonia, Brazil^25^. Most of the published data focused either on paediatric populations^26^ or on specific vaccines^27^.

The main aim of this study was to describe and analyse the Italian PhV system on vaccines in the last 10 years. Specific objectives were: (i) to describe and analyse the Italian AEFI reporting rate (within the EU context), between 2008 and 2017, (ii) to present a detailed overview of all reported AEFIs and (iii) to provide a brief overview of main signals detected during the study period.

## Methods

### Data source: Italian pharmacovigilance system and database

The Italian PhV System is based on a network connecting the Italian Medicines Agency (*Agenzia Italiana del Farmaco* [AIFA]) to 21 regional authorities, including the Regional PhV Centers (RPC), the local health authorities/hospitals/research institutes, the local PhV representatives (LRP), and pharmaceutical companies. The AIFA manages post-marketing drug and vaccine surveillance. RCPs collaborate with the AIFA to monitor adverse drug reactions and detect safety signals. They cooperate with the prevention measures implemented in vaccinovigilance programmes. Adverse reaction reports were collected and analyzed through the National PhV Database (RNF), which was created in 2001. Most of the RNF data consist of spontaneous reports of suspected adverse reactions made by health care professionals (HCPs), patients, and active surveillance projects. Spontaneous reports are transmitted to LRPs in hard copy form or through a web-based system (Vigifarmaco). AEFIs, laboratory tests, and therapeutic indications are coded according to the Medical Dictionary for Regulatory Activities (MedDRA®)^28^.

All reports are transferred to the European database EudraVigilance managed by the European Medicines Agency (EMA), and to the World Health Organization (WHO) global database (Vigibase).

### Data extraction and analysis

AEFI reports from 2008–2017 were extracted from the RNF database. The annual reporting rate (RR) per distributed doses was calculated both overall and per reports with at least one serious AEFI (denominator available since 2009). Moreover, the number of reports from other countries in the same period was obtained from the global database Vigibase. The reporting rate per 1,000,000 inhabitants was calculated for Italy and other EU countries (denominators calculated from Eurostat database)^29^.

All AEFIs collected by the RNF in the study period were categorized according to primary source, age of vaccine administration, outcome, and seriousness according to the definitions in DIR 2001/83/EC Art 1^30^ and the Important Medical Event (IME) List^31^.

AEFI reports are grouped according to five age classes: infants (< 23 months), children (2–11 years), adolescents (12–17 years), adults (18–64 years), and elderly (> 65 years).

A report is defined as serious when at least a serious adverse reaction is reported.

Serious adverse reaction is defined as “any untoward medical occurrence that at any dose results in death, is life-threatening, requires inpatient hospitalization or prolongation of existing hospitalization, results in persistent or significant disability or incapacity, or is a congenital anomaly/birth defect”^30^. An AEFI may be deemed serious according to medical judgement if a clinically relevant event is present. EMA developed a list of IME Terms to facilitate the classification of clinically relevant events^31^.

All reports with at least a serious event are evaluated for the likelihood of a causal relationship between the vaccine and the reported AEFI. Causality is assessed by the RCPs and the AIFA and, since 2015, is evaluated in accordance with the new tool developed by WHO, classifying reports as consistent, inconsistent, indeterminate and unclassifiable^32,33^.

All collected AEFIs are regularly evaluated for signal detection activity. Signal detection is carried out according to a specific standard operating procedure, including disproportion analysis with the Reporting Odds Ratios and compliance with European provisions and guidelines like such as Good Pharmacovigilance Practices^34^.

## Results

### AEFI reporting rates

A total of 46,430 reports were submitted between 2008 and 2017. They referred to 63,457 suspected vaccines and described 96,926 AEFIs. The mean number of AEFIs per report was 2.1.

**Figure 1:**
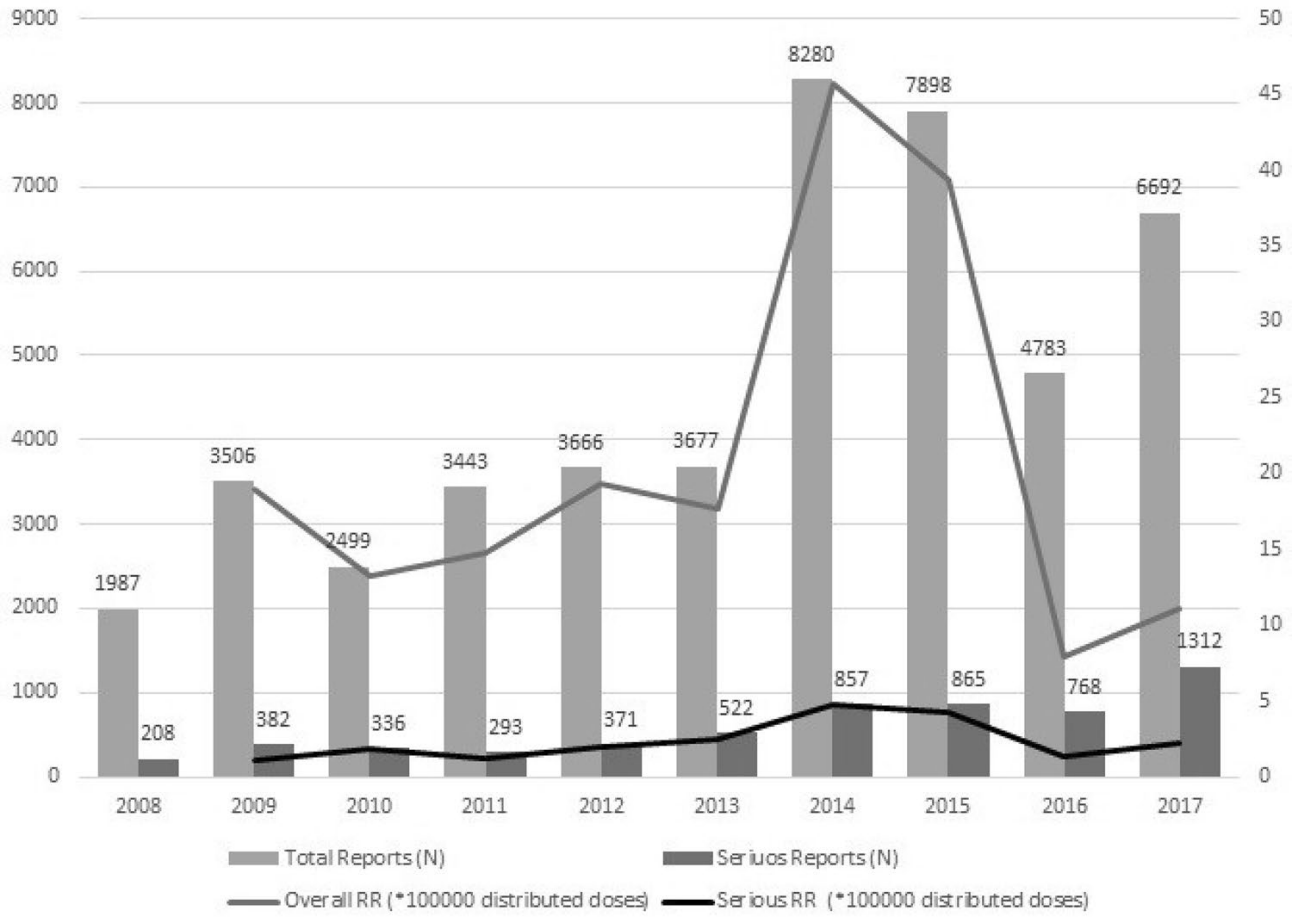
Distribution (N) of reports and Reporting Rate per distributed dose of AEFI by year, from 2008 to 2017.

Figure 1 shows the annual RR per distributed dose both overall and per report with at least a serious event (denominator available since 2009). The overall trend shows an increase with time and three peaks in 2009, 2014–15, and 2017 while RR per serious reports show a lower variability over time ranging from 1.3 (2003) to 2.2 (2017) with two peaks in 2014 (4.7) and 2015 (4.3).

The increase in 2014 and 2015 is related to the doubled proportion of cases of hyperpyrexia in comparison to previous years. Considering the whole study period, overall RR and RR per reports with at least one serious AEFI were respectively 17.1 and 2.2 per 100,000 distributed doses. More than 770,000 vaccine related reports were sent to the WHO Vigibase during this decade. Most of them originated in the US (57.2%) and Europe (29.0%). Italy had the second-highest absolute number of reports worldwide after the US. Among the European countries, Italy accounted for the highest number of reports (19.4%), followed by Germany (17.4%), the Netherlands (10.2%), France (8.9%), the UK (8.4%), and Spain (6.9%).

**Figure 2:**
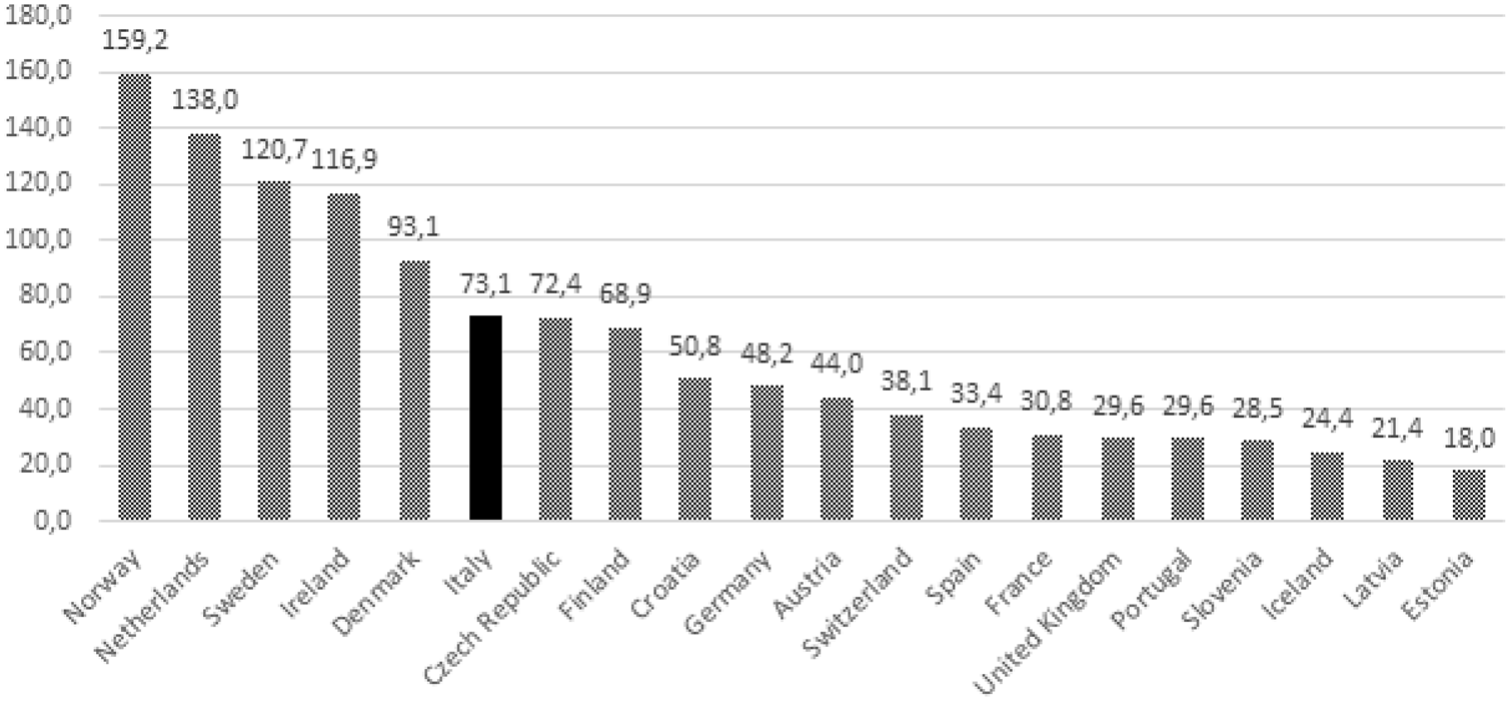
Reporting rate of AEFI per 1,000,000 inhabitants by EU Country among the study period (2008–2017).

Figure 2 shows the AEFI RR per 1,000,000 inhabitants by EU country. The 20 countries with the highest reporting rates from 2008 to 2017 were considered.

### General report characteristics

Almost all reports (95.6%) had the most important information needed for a causality assessment evaluation, including time of onset of the reaction, date of vaccine administration, reporter role, age group, gender, and seriousness based on the seriousness criteria. Moreover, all reports with the most serious adverse events had not only the AEFI description, but also further narratives of the whole case written by the reporter, with the attachment of detailed documents such as hospital clinical charts.

Table 1 shows the distribution of reports according to the source. A large number of reports came from doctors: among these, most worked in vaccination centres (50%), 15% worked in hospitals, 5,9% were general practitioners and 5,3% were paediatricians. Other health operators include nurses and health care assistants at the vaccine centres. The number of reports from citizens was significantly higher in 2017 than the previous years (889 reports in 2017, 43% of total reports from citizens). More than half of these referred to events which occurred in earlier years.

**Table 1:** Distribution of reports by source.

Source	N. of reports	%
Doctors	32,233	69.4
Pharmacists	2,148	4.6
Other health operators	9,928	21.4
Citizens	2,054	4.4
Not available	67	0.1
Total	46,430	100.0

Table 2 shows the distribution of reports according to age groups. The highest part of reports are related to infants. The proportions of serious AEFIs varied among the different age classes, with the highest value in the elderly.

**Table 2:** Distribution of reports by age group with percentages of serious reports.

Age group	N. of reports	%	% serious
Infants (< 23 months)	25,863	55.7	13.5
Children (2–11 years)	9,115	19.6	9.6
Adolescents (12–17 years)	3,051	6.6	11.6
Adults (18–64 years)	5,747	12.4	9.8
Elderly (> 65 years)	2,466	5.3	24.0
Not reported	188	0.4	19.7
Total	46,430	100.0	12.7

### Description of reports with at least a serious AEFI

Of the 46,430 reports collected for the decade, 5,914 (12.7%) were classified as serious. Among these, 2,794 (47.2%) were cases requiring hospitalization (including ER admission) or where hospitalization was prolonged. Another 2,617 (44.3%) referred to other relevant events. Causality assessment evaluation of reported adverse events has been performed for reports with at least a serious event. Since 2015, when the new tool for causality assessment developed by the WHO was used, only 7.7% of reports lacked adequate information and were categorized as unclassifiable. The majority of serious reports were considered consistent with the vaccination (64.0%), 11.1% were indeterminate and 17.2% were inconsistent. The proportion of both unclassifiable and inconsistent reports increased from 2015 to 2017, mainly due to reports from patients. The most frequently reported serious reactions in inconsistent reports were hyperpyrexia and febrile convulsions (due to unrelated time of onset) and autism spectrum disorders. Outcome data were available for 90.9% (42,184/46,430) of the reports. Of these, 84% recovered and 11.2% improved. Fatal outcomes were reported for 0.3% of the total AEFIs (N = 129). None of these outcomes in the causality assessment showed a consistent causal association with vaccination. More than 80% of fatal reports involved influenza vaccines administered to the elderly adults.

Table 3 shows the distribution of AEFIs by most commonly reported vaccine type and the proportion of serious reported events.

**Table 3:** Distribution of AEFIs with > 500 reports per vaccine type, and AEFI seriousness.

Vaccine	Total AEFIs (N)	Serious AEFIs (%)
DTaP-HB-IPV-HIB	10,429	13.7
PNEUMO-con13	7,950	12.7
MMR	7,909	13.4
MMRV	5,375	14.3
HPV	5,243	5.9
VAR	4,589	9.3
MENB	3,967	13.0
FLU	2,587	21.8
DTaP-IPV	2,443	8.3
MENC-con	1,968	18.0
PNEUMO-con	1,409	14.9
FLU_H1N1	1,356	8.0
MEN_4-con	1,122	16.8
DT	844	7.9
DTaP	820	13.8
PNEUMO	763	9.8
DTP	713	13.6
HA	677	14.9
TT	563	9.4
FLU_ad	524	31.5
ROTAVIRUS	524	16.8

DTaP-HB-IPV-HIB: diphtheria, tetanus, acellular pertussis, hepatitis B, inactivated polio, and *Haemophilus influenzae* type b; PNEUMO-con13: pneumococcal conjugate vaccine (13-valent); MMR: measles, mumps & rubella; MMRV: measles, mumps, rubella & varicella; HPV: human papillomavirus vaccine; VAR: varicella vaccine; MENB: serogroup B meningococcal vaccine; FLU: flu vaccine; DTaP-IPV: diphtheria, inactivated polio; MENC-con: meningococcal C conjugate vaccine; PNEUMO-con: pneumococcal conjugate vaccine; FLU_H1N1: flu H1N1 vaccine; MEN_4-con: meningococcal conjugate vaccine, quadrivalent; DT: diphtheria, tetanus; DTaP: diphtheria and tetanus toxoids and acellular pertussis vaccine; PNEUMO: pneumococcal vaccine; DTP: diphtheria and tetanus toxoids and whole-cell pertussis vaccine; HA: hepatitis A virus vaccine; TT: tetanus vaccine; FLU_ad: influenza vaccine, adjuvanted.

Often more than one vaccine is administered to the same person during the same vaccination session. The most commonly reported vaccine combinations were: hexavalent + pneumococcal conjugate 13 (PCV-13) (6,082/46,430, 13.1%), MMR + V (3,929/46,430, 8.5%) and hexavalent + pneumococcal conjugate (PCV7) (1,030/46,430, 2.2%). The vaccines with the highest proportion of serious events were INF (21.8%) and INF-ad (31.5%). Table 4 shows the most frequently reported AEFIs according to MedDRA Preferred Terms.

**Table 4:** List of the most frequently reported AEFIs (> 500).

MedDRA PT	MedDRA SOC	Reports (N)
Pyrexia	General disorders and administration site conditions	16,763
Hyperpyrexia	General disorders and administration site conditions	5,165
Vaccination site pain	General disorders and administration site conditions	2,898
Headache	Nervous system disorders	2,741
Post-vaccinal Irritability	General disorders and administration site conditions	2,687
Morbilliform rash	Skin and subcutaneous tissue disorders	2,494
Pain	General disorders and administration site conditions	2,161
Injection site erythema	General disorders and administration site conditions	2,083
Vaccination site swelling	General disorders and administration site conditions	2,039
Somnolence	Nervous system disorders	1,835
Erythema	Skin and subcutaneous tissue disorders	1,809
Rash	Skin and subcutaneous tissue disorders	1,679
Crying	General disorders and administration site conditions	1,515
Urticaria	Skin and subcutaneous tissue disorders	1,499
Vomiting	Gastrointestinal disorders	1,470
Vaccination site reaction	General disorders and administration site conditions	1,376
Irritability	Psychiatric disorders	1,295
Cough	Respiratory, thoracic and mediastinal disorders	1,228
Nasopharyngitis	Infections and infestations	1,187
Local reaction	General disorders and administration site conditions	1,096
Diarrhea	Gastrointestinal disorders	1,094
Myalgia	Musculoskeletal and connective tissue disorders	1,079
Injection site pain	General disorders and administration site conditions	1,062
Asthenia	General disorders and administration site conditions	1,048
Restlessness	Psychiatric disorders	1,011
Arthralgia	Musculoskeletal and connective tissue disorders	989
Nausea	Gastrointestinal disorders	949
Flushing	Vascular disorders	921
Agitation	Psychiatric disorders	906
Pruritus	Skin and subcutaneous tissue disorders	853
Decreased appetite	Metabolism and nutrition disorders	824
Pain in extremity	Musculoskeletal and connective tissue disorders	754
Lymphadenopathy	Blood and lymphatic system disorders	700
Swelling	General disorders and administration site conditions	605
Edema	General disorders and administration site conditions	570
Malaise	General disorders and administration site conditions	559
Abdominal pain	Gastrointestinal disorders	556
Hypotonia	Nervous system disorders	548
Vaccination site edema	General disorders and administration site conditions	504
Febrile convulsion	Nervous system disorders	502

There were 8,831/96,731 reported IMEs (9.1%). The most frequently reported IMEs were hyperpyrexia, seizures, and loss of consciousness. Various types of seizures/epilepsy have been reported along with fever (pyrexia or hyperpyrexia) and described as febrile convulsions. There were 502 cases reported as febrile convulsions. Another 227 cases presented with these symptoms but were described using a combination of terms related to both seizures and fever. Sixty-five reports described autism spectrum disorders. These were deemed unrelated to the vaccination in the causality assessment.

### Most significant safety signals

During the study period, all potential signals were routinely and closely investigated and analysed. In most cases, the potential signals were not confirmed during the validation procedure. However, when needed, monitoring was integrated with active surveillance by conducting epidemiological studies. The most relevant safety signals in the study period included the risk of febrile seizures related to measles, mumps, rubella, varicella (MMRV) vaccine, neurological events and convulsions related to the co-administration of 13-valent pneumococcal polysaccharide conjugate (PCV13) and hexavalent vaccines, Guillain–Barré Syndrome (GBS) after influenza vaccination and intussusception after rotavirus vaccine. Finally, no reports of postural orthostatic tachycardia syndrome (POTS) or complex regional pain syndrome (CRPS) were identified following HPV vaccination.

## Discussion

Due to the known limitations of the pre-marketing clinical trials, the spontaneous reporting systems are the cornerstone of post-marketing drug safety surveillance^35^. Spontaneous reporting is essential in signal detection and the identification of new risks associated with drugs, but can also be very useful in the evaluation of the safety profile of both drugs and vaccines^36^. There are no globally accepted indicators which could demonstrate the functionality of an AEFI surveillance system. However, underreporting and incomplete reports are the most important limitations of spontaneous reporting^37,38^, so a high number of reports and a high good quality of the reported information are clearly related to the strength of the system. Moreover, a strong collaboration between the drug regulatory agency and the national immunization programme and the availability of reliable information on the actual number of immunizations have both been reported as essential components of an efficient surveillance system of AEFIs^39^.

The absolute number of reports in Italy is the highest one in Europe and the worldwide second after that the US. Despite this huge amount of information, up to our knowledge this is the first attempt to provide an overview of Italian vaccinovigilance safety data.

The numbers show an increasing trend since 2008, in line with what has been observed in many other countries^40,41^. Of all European nations, Italy had highest number of reports among European countries with at least 10 million inhabitants.

The increase in reports is the result of the combined global efforts to improve vaccinovigilance systems.

In Italy, the collaboration between the AIFA and the RPCs promoted reporting activity substantially using various strategies such as enhancements endorsed at the policy level, interventions to stimulate AEFI reporting in vaccination districts, and the development of health care education and training programmes. Since 2009, the AIFA committed to disseminating vaccinovigilance safety data to ensure transparent management of emerging issues and to favour the diffusion of accurate information to the public. Italy submitted the most AEFIs of all European countries to the WHO during the survey period, indicating that the Italian vaccinovigilance system has expanded over time. The number of reports and the reporting rates related to population or to administered doses are very useful as performance indicators of the system to monitor the trends over time. However, they do not provide information on the quality of the system, particularly on the ability of the system to detect vaccine safety problems^40^.

According to our results, AEFI reports were of high quality, allowing for the analysis of a large amount of detailed information. A study by the WHO reported that Italy had the highest rate of well-documented reports (65%) among nations submitting > 1,000 reports per year^42^. The observed data quality, together with the ability of the system to detect serious AEFIs, supports the reliability of the results.

The peaks in the observed Italian AEFI reporting rates per 100,000 distributed doses correlate with those of active surveillance programmes complementing passive monitoring systems^43–45^. They help to raise awareness towards specific potential safety issues and increase the attention paid to new vaccines when market experience is still limited (e.g. HPV or MenB introduction).The 2009 peak in AEFI reporting may be explained by the introduction of the HPV vaccine in 2008 and monitoring of the H1N1 cases during the flu pandemic.

The AEFI reporting peak observed in 2017 may be associated with the increase in the number of compulsory vaccinations from 4 to 10 mandated by Law No. 119/2017 enforced in July 2017^46^. Moreover, in 2017 the National Vaccinating Plan^47^ expanded the scope of free vaccinations. The changes contributed to increasing the AEFI RR in 2017.

Reporting rates per administered doses were not calculated due to unavailability of the data along the study period in Italy. Efforts focus on developing and implementing computerized immunization registries in as many regions as possible. The comparison between reporting rates and background rates of the diseases is very important in AEFI detection. Background rates of possible adverse events are a crucial part of the assessment of possible vaccine safety concerns and help to separate causally related events from those only temporally associated with but not caused by vaccination^48,49^.

A survey conducted in 2012 showed that only six of the 15 fully computerized regions could automatically calculate vaccination coverage^50^. This number increased to 9 of the 18 fully computerized on 21 total regions in 2016^51^. The regional variability can be explained by the decentralization of the Italian health care system^51^. However, Law No. 119 of July 31, 2017 ordered the implementation of a national immunization registry at the Ministry of Health. To improve the relationship between the Drug Agency and the Public Health Authority, in 2014, a Vaccinovigilance Working Group (VWG) was created by the AIFA. It included the National Institute of Health, the Minister of Health, and both PhV and public health members. The main aim of this group was to contribute to signal detection activities.

More than half of the AEFIs originated either from doctors or other health care professionals. Patients have the lowest reporting frequency of all groups. Patient reporting may play an important role in pharmacovigilance. Patients not only contribute to reducing the underreporting of adverse reactions, but the quality of their reports is similar to the quality of healthcare operators. In comparison to doctors and pharmacists the description of the reported adverse reactions is often more detailed with a different perspective^52–54^. Patient empowerment projects improve the efficacy of a passive surveillance system and facilitate the creation of a trusting relationship that dispels false beliefs and allays fears regarding vaccination.

In 2013, a project to stimulate AEFI reporting from both patient and health care operators significantly increased the reporting rate per 100,000 administered vaccine doses (from a mean of 1,7 per 100,000 administered doses in 2009–2012 to approximately 14.0 per 100,000 in 2013 after implementing the intervention)^55^. The lower variability of RR per reports with at least a serious event compared with RR per administered doses suggests a limited underreporting for serious AEFIs. Approximately 13% of the reports included at least a serious AEFI. This percentage is higher in comparison to other published data^56–58^ and could suggest a higher risk of serious events associated to vaccinations. However, all serious AEFIs must be closely investigated to establish any causal relationship with vaccine administration.

Nearly all cases reported as serious either required hospitalization/prolonged hospitalization or experienced important medical events. Together, they accounted for > 90% of all reported AEFIs. However, hospitalized cases included also those treated in the ER and discharged without admission, because the reported event was resolved.

Among the important medical events, one of the most frequently reported serious AEFIs was high fever which accounted for almost 2/3 of the total reported IME. It is a relatively common adverse event, but not usually dangerous. The second most commonly reported event was seizure. Nevertheless, most of these cases were associated with hyperpyrexia. According to the CDC, febrile seizures are fairly common, with a lifetime prevalence ≤ 5%. Nevertheless, febrile seizures rarely occur after vaccination^59^. The proportion of reported AEFIs that were rated serious was significantly higher among the elderly adults than among those in other age groups. This age group includes a higher percentage of frail people including patients suffering from chronic diseases or other medical conditions. Therefore, this age group may substantially increase the total rate of reported serious events including those not attributable to the vaccines.

Approximately 2 out of 3 of the fatal cases were adults or elderly patients, who had received influenza vaccine. Most of these reports were posted in 2014 and were related with the prudent withdrawal of two batches of conjugated flu vaccine (trivalent influenza vaccine with MF59C.1 adjuvant) after three post-vaccination deaths were reported^60^. After the evaluation by EMA the two flu vaccine batches were reintroduced, since none of the reported deaths had any consistent causal relationship with the flu vaccine.

The reporting rate of serious report was quite stable over time indicating the capacity of the system to detect safety issues. The highest incidences of AEFI reporting occurred for the most frequently administered vaccines, namely, hexavalent, pneumococcus, MMR, and MMRV. The high number of AEFIs reported for HPV can be explained by active surveillance. For this vaccine, even minor reactions and incidents were reported to the RNF. Compared with the other vaccines, HPV accounted for only a small proportion of the reported serious events. Flu vaccines were associated with the highest proportion of serious reported events (≤ 1/3 of all reports for MF59 adjuvanted flu vaccine). The most frequently reported AEFIs included fever, pain, asthenia, injection site reactions, rash, allergic reactions, headache, drowsiness, and febrile seizures.

 The main goal of spontaneous reporting systems is to identify signals, and during the study period, all potential signals were closely investigated and analysed. The international literature was reviewed to evaluate each signal. Moreover, formal studies were conducted to investigate the most significant signals. The most important signals in the study period included the following:

- Higher risk of febrile seizures with the measles, mumps, rubella and varicella (MMRV) vaccine compared with separate vaccination with measles, mumps and rubella (MMR) and varicella. Product information of the involved vaccines was updated, and a communication was sent to all the health professionals^61^
- Risks of neurological events and convulsions after the co-administration of 13-valent pneumococcal polysaccharide conjugate (PCV13) and hexavalent vaccines. A comparative safety evaluation of 7-valent and 13-valent pneumococcal vaccines in routine paediatric vaccinations was conducted in four Italian regions from 2009 to 2011^62^. A slightly increased risk of neurological events or convulsions was found but, due to the study limitations, no regulatory decision was taken.
- Risk of Guillain–Barré Syndrome (GBS) after influenza vaccination. A case–control study was conducted in 2010 in seven regions of Italy to explore this correlation (GBS). Influenza vaccination was associated with GBS, with a relative risk of 2.1 (95% CI 1.1, 3.9). The results of the study did not modify the risk–benefit profile of seasonal influenza vaccination. The attributable risk in adults ranged from two to five GBS cases per 1,000,000 vaccinations^63,64^
- Risk of intussusception associated with the administration of rotavirus vaccines. A study was conducted to investigate the representative intussusception incidence background in the different age groups and its temporal trend in Italy^65^. The overall intussusception incidence rate was 21 per 100,000 children aged ≤ 15 years and higher among boys than girls.

### Strength and limitations

To our knowledge, no studies have been published on global Italian vaccinovigilance data. Previous literature presented data either at the regional level or for the paediatric population. No comprehensive view of Italian vaccinovigilance activity or its evolution over time has yet been reported. The strength of this study is to fill in this information gap and make data available for international comparison. A limitation of the present study is that the AEFI reporting rates cannot be related to the actual number of administered doses. Another limitation concerns the timing of the report updates. In the present study, the AEFIs were analysed according to the date of receipt of reports by the RNF and not by the date of vaccine administration. This could have an impact on the reporting rate due to the delayed reporting.

### Conclusion

This report is an overview of the Italian vaccinovigilance data. Over the past decade, the system has allowed for the detection of a large amount of reliable data, including information regarding serious AEFIs. About ten multicentre active surveillance projects were conducted to collect and monitor a larger amount of safety-related data. Timely analyses of all signals were performed across the nation. The reporting rate and the analysis of serious reports from 10 years of AEFIs support the risk/benefit profiles of vaccines. The low participation rate of citizens in vaccine safety management emerged as an important issue. The quality of the data is strictly related to the availability of precise denominators (most importantly the number of administered doses or at least the number of distributed doses). Further efforts need to be implemented to have national vaccine registries and to link this information with surveillance data. Transparency, information, and population empowerment on immunization safety must be promoted through effective communication.
